# [^13^C]bicarbonate labelled from hyperpolarized [1-^13^C]pyruvate is an in vivo marker of hepatic gluconeogenesis in fasted state

**DOI:** 10.1038/s42003-021-02978-2

**Published:** 2022-01-10

**Authors:** Emine Can, Jessica A. M. Bastiaansen, Dominique-Laurent Couturier, Rolf Gruetter, Hikari A. I. Yoshihara, Arnaud Comment

**Affiliations:** 1 Institute of Physics, Ecole Polytechnique Fédérale de Lausanne, CH-1015 Lausanne, Switzerland; 2 Department of Diagnostic and Interventional Radiology, Lausanne University Hospital and University of Lausanne, Lausanne, Switzerland; 3 Cancer Research UK Cambridge Institute, University of Cambridge, Cambridge, Cambridgeshire CB2 0RE UK; 4 General Electric Healthcare, Chalfont St Giles, Buckinghamshire, HP8 4SP UK

**Keywords:** Hepatocytes, Preclinical research, Metabolomics, Magnetic resonance imaging

## Abstract

Hyperpolarized [1-^13^C]pyruvate enables direct in vivo assessment of real-time liver enzymatic activities by ^13^C magnetic resonance. However, the technique usually requires the injection of a highly supraphysiological dose of pyruvate. We herein demonstrate that liver metabolism can be measured in vivo with hyperpolarized [1-^13^C]pyruvate administered at two- to three-fold the basal plasma concentration. The flux through pyruvate dehydrogenase, assessed by ^13^C-labeling of bicarbonate in the fed condition, was found to be saturated or partially inhibited by supraphysiological doses of hyperpolarized [1-^13^C]pyruvate. The [^13^C]bicarbonate signal detected in the liver of fasted rats nearly vanished after treatment with a phosphoenolpyruvate carboxykinase (PEPCK) inhibitor, indicating that the signal originates from the flux through PEPCK. In addition, the normalized [^13^C]bicarbonate signal in fasted untreated animals is dose independent across a 10-fold range, highlighting that PEPCK and pyruvate carboxylase are not saturated and that hepatic gluconeogenesis can be directly probed in vivo with hyperpolarized [1-^13^C]pyruvate.

## Abstract (web summary)

Can et al. demonstrate the ability to use hyperpolarized [1-13C]pyruvate at nearphysiological concentrations to directly assess liver enzymatic activities by 13C magnetic resonance. While in the fed state, the normalized [13C]bicarbonate signal produced from hyperpolarized [1-13C]pyruvate derives from PDH activity, which is saturated at supraphysiological doses, it results from PEPCK in the fasted state and is dose-independent, allowing non-invasive in vivo detection of hepatic gluconeogenesis.”

## Introduction

**Fig. 1:**
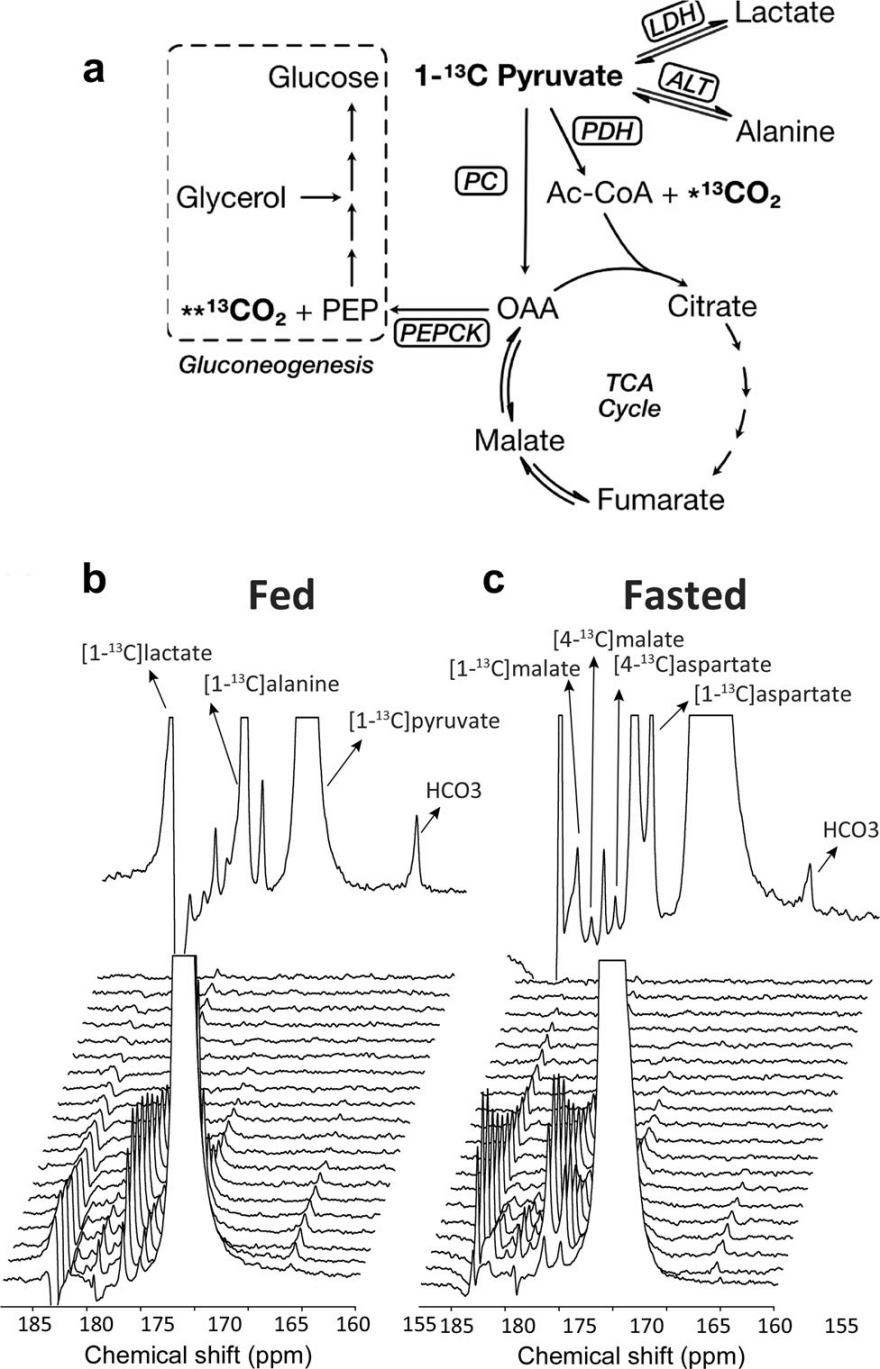
Carbon flow from [1-^13^C]pyruvate through TCA cycle and GNG in liver and representative evolution of the ^13^C MR spectra measured in the rat liver. **a** ^13^C labeling of injected pyruvate is converted to C1 of lactate and alanine through lactate dehydrogenase (LDH) and alanine aminotransferase (ALT), respectively. Decarboxylation of [1-^13^C]pyruvate through pyruvate dehydrogenase (PDH) yields ^13^CO_2_ and acetyl-CoA. The released ^13^CO_2_ is interconverted with bicarbonate (H^13^CO_3_) through carbonic anhydrase. Pyruvate carboxylase (PC) converts [1-^13^C]pyruvate to [1-^13^C]oxaloacetate (OAA). C4 of OAA is labeled after conversion to fumarate and 1,4-label scrambling. OAA contributes to citrate formation in condensation with acetyl-CoA and is also converted to phosphoenolpyruvate (PEP) through phosphoenolpyruvate-carboxykinase (PEPCK). PEP contributes to the production of glucose via an eight-step pathway. 3-mercaptopicolinic acid (3-MPA) inhibits glucose synthesis by blocking conversion of OAA to PEP. *^13^CO_2_ indicates labeled CO_2_/bicarbonate from PDH flux; **^13^CO_2_—from PEPCK flux; Spectra in fed (**b**) and fasted (**c**) animals were measured every ~3 s following the injection of 0.023 ± 0.002 mmol/kg HP [1-^13^C]pyruvate in the femoral vein. The summed ^13^C MR spectra are also shown.

Homeostatic control of blood glucose level in response to nutritional conditions, such as fasting hypoglycemia or postprandial hyperglycemia, is an essential role of the liver. It is maintained by the storage of glucose as glycogen polymers, which are in turn broken down to release glucose as needed, as well as by the synthesis of glucose (gluconeogenesis (GNG)) from non-carbohydrate substrates, particularly glycerol, alanine, lactate, and pyruvate^1^. The latter lies at a crossroads of glucose metabolism since it can be used as a substrate in both catabolic and anabolic processes in the liver (see Fig. 1a). The conversion of pyruvate to glucose begins with the carboxylation of mitochondrial pyruvate to oxaloacetate (OAA) by pyruvate carboxylase (PC) and subsequent decarboxylation of OAA to phosphoenolpyruvate (PEP) through PEP-carboxykinase (PEPCK) which initiates GNG^2^. Pyruvate can also enter the mitochondria to feed the tricarboxylic acid (TCA) cycle through a reaction catalyzed by pyruvate dehydrogenase (PDH). The flux of pyruvate through these two parallel pathways can be difficult to independently quantify because the TCA cycle intermediate OAA is also an intermediate of gluconeogenic metabolism.

The endocrine and biochemical mechanisms controlling GNG have been of great interest because their dysregulation is implicated in critical pathologic conditions such as diabetes, fatty liver disease, and cancer development^3–6^. The rates of GNG and related pathways depend on several factors, such as transcriptional regulation of enzyme expression, changes in substrate flux, fatty acid oxidation and the TCA cycle activity that provides the supply of mitochondria-derived substrates and adenosine triphosphate (ATP) needed to run GNG^6^. Being able to noninvasively probe GNG in vivo would provide a way to better understand and assess the efficacy of treatments for diseases such as type 2 diabetes that target hepatic glucose metabolism^7^.

Gluconeogenic and glycolytic metabolism has been investigated in blood and urine sample from rodents^8,9^ and humans^10–12^ by ^13^C nuclear magnetic resonance (NMR) following the administration of various ^13^C-labeled substrates. Although a 2-h infusion of [1-^13^C]acetate and a complex mathematical model allowed Befroy et al. to estimate gluconeogenic fluxes from noninvasive in vivo ^13^C magnetic resonance spectroscopy (MRS) measurements^13^, such measurements are challenging in the liver due to their low sensitivity and the natural-abundance ^13^C lipid resonances impairing the detection of the labeled ^13^C-glutamate signal from which fluxes are determined. Alternatively, ^13^C-labeled substrates can be hyperpolarized (HP) by dynamic nuclear polarization (DNP) prior to intravenous administration, and the dramatically enhanced ^13^C MRS signal intensities enable real-time tracking of their metabolic conversion^14^ with minimal interference from endogenous ^13^C signals. Intermediary metabolism can be noninvasively probed in vivo using HP ^13^C-pyruvate and this unique method has already been translated to humans^15,16^. The administration of HP ^13^C-pyruvate enables the direct measurement of real-time liver mitochondrial enzymatic activities related to the gluconeogenic processes via the incorporation of ^13^C label into metabolite pools (see Fig. 1a). In order to obtain a sufficient metabolite signal, HP ^13^C pyruvate is typically injected at doses ranging from 0.1 to 1 mmol/kg. This results in blood pyruvate levels far in excess of the normal level, and the observed metabolic fluxes may be altered by this supraphysiological condition.

Following the injection of HP [1-^13^C]pyruvate in the perfused mouse liver, Merritt et al. showed that the HP [^13^C]bicarbonate detected results from PEPCK activity, and the TCA cycle intermediates malate and aspartate represent a reading of PC^17^. However, subsequent in vivo HP ^13^C measurements performed in the rat liver together with ex vivo non-HP ^13^C NMR experiments led to the conclusion by Jin et al. that the [^13^C]bicarbonate signal mostly originates from PDH activity in well-fed animals^18^. The same study also reported that no [^13^C]bicarbonate signal could be detected in fasted animals despite also showing with ex vivo non-HP ^13^C NMR experiments that nearly all the injected pyruvate flows through PC in this nutritional state. Unlike Jin et al., several research groups observed a [^13^C]bicarbonate signal in vivo in the liver following the injection of HP ^13^C-pyruvate in fasted rodents^19–21^, albeit at lower levels compared to the fed state. All these other in vivo studies concluded however that the [^13^C]bicarbonate signal mainly originates from PDH activity.

This study aimed to determine the relative contributions of PDH and PEPCK activity to the liver [^13^C]bicarbonate signal in vivo and to assess the effect of HP pyruvate dose on the metabolic response in order to define the useful dosage range with minimal metabolic perturbation.

## Results

### Hepatic metabolism of HP [1-^13^C]pyruvate at near-physiological concentrations

The semi-automated setup (see Methods section) used for the in vivo experiments provided a reliably high ^13^C polarization (60 **±** 5%) for all injected HP [1-^13^C]pyruvate doses, leading to consistent observation of the metabolic products [^13^C]bicarbonate, [1-^13^C]alanine, [1-^13^C]lactate as well as [1-^13^C]aspartate, [4-^13^C]aspartate, [1-^13^C]malate, and [4-^13^C]malate. A respiration-gated ^13^C spectrum was recorded every ~3 s following the injection of HP [1-^13^C]pyruvate and all data analyses were performed on the sum of ~15 consecutive individual spectra (Fig. 1a, b). This way, the smaller malate and aspartate signals could still be accurately quantified at the lowest dose tested (0.023 **±** 0.002 mmol/kg). This dose results in an estimated maximum plasma level of 0.35 **±** 0.03 mM just after injection, which is only two- to three-fold the basal level in Sprague Dawley rats^22^. Because the absolute metabolite concentrations cannot be directly extracted from the HP ^13^C spectra due to the uncertainty in the remaining in vivo signal enhancement resulting from hyperpolarization, it is common practice to normalize each metabolite signal to the total ^13^C signal (see Figs. S1 & S2). However, since the measured ^13^C pyruvate signal in the present study originates almost exclusively from the blood pool, while the ^13^C signal from the downstream metabolic products is largely intracellular, we instead chose to base our analyses on the individual ^13^C metabolite signals normalized by the total ^13^C signal for all metabolites, excluding the ^13^C substrate signal from the denominator. This is an approach that has already been proposed^23^, and in many studies with HP [1-^13^C]pyruvate, an analysis based on the ratio between two metabolites (e.g. bicarbonate/lactate^24^ or lactate/alanine^25^) is preferred to reduce any variability due to the mismatch between the HP signals of [1-^13^C]pyruvate in the blood and its metabolites in tissue.

### Effect of dose on hepatic metabolism of HP [1-^13^C]pyruvate

**Fig. 2:**
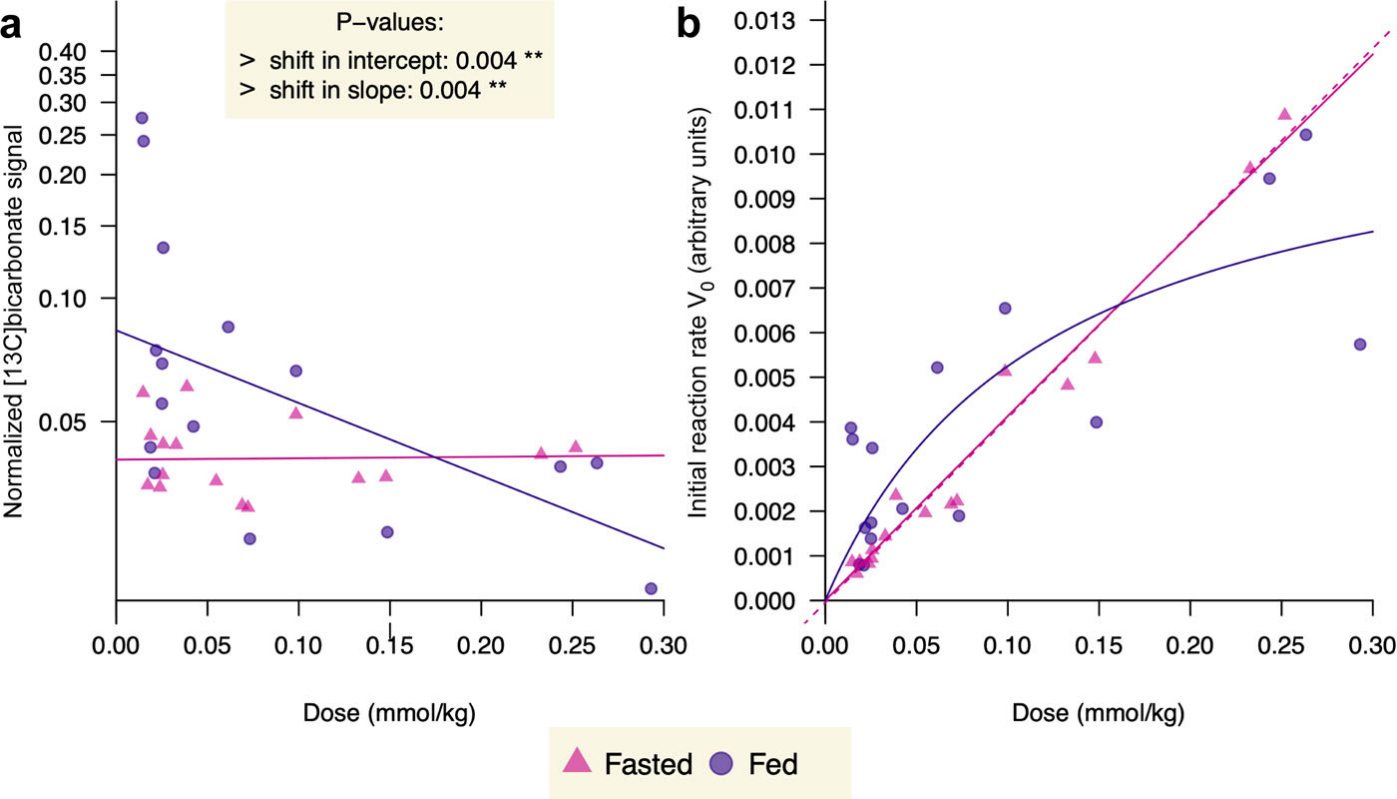
Scatter plot of the HP [^13^C]bicarbonate signal intensities normalized to the total sum of metabolites and corresponding initial reaction rate as a function of the injected dose of HP [1-^13^C]pyruvate. Points for 32 experiments (16 fed; 16 fasted) are coded by nutritional state (purple circles: fed; pink triangles: fasted). **a** The fitted regression line of a heteroscedastic linear model is shown for each state. The *p*-values correspond to the two-sided Wald t-tests of equality of intercept and slopes. **b** The initial reaction rate *V*_0_ (in arbitrary units) was obtained by multiplying the normalized HP [^13^C]bicarbonate signal, which can be assumed to be proportional to an apparent pyruvate-to-bicarbonate conversion rate constant (*k*_pyr-bic_), with the corresponding substrate dose. The dotted line is a linear regression.

The variations in normalized metabolites signals recorded as a function of the injected pyruvate dose were analyzed using linear models for both nutritional states, yielding a slope and an intercept for each state (Fig. 2a). Increasing the amount of HP [1-^13^C]pyruvate infused up to 0.29 mmol/kg, revealed a clear dose-dependent difference in [^13^C]bicarbonate production between the fed and overnight-fasted rats. Whereas the normalized bicarbonate signal did not vary with dose in fasted rats, it decreased with higher doses in fed rats, and linear fits of dose versus the log-scaled bicarbonate signal show significantly different slopes and y-intercepts. To highlight the saturation in [1-^13^C]pyruvate to [^13^C]bicarbonate conversion rate observed in the fed state, an additional analysis with the more intuitive and familiar Michaelis-Menten model was performed using an approach similar to Zierhut et al.^26^ and Bastiaansen et al.^27^ (Fig. 2b). The fit yields a *K*_m_ value of 0.12 **±** 0.075 mM for the fed state and *K*_m_ **=** 15 **±** 25 mM for the fasted state, the latter being clearly better fit with a linear regression (dotted line).

**Fig. 3:**
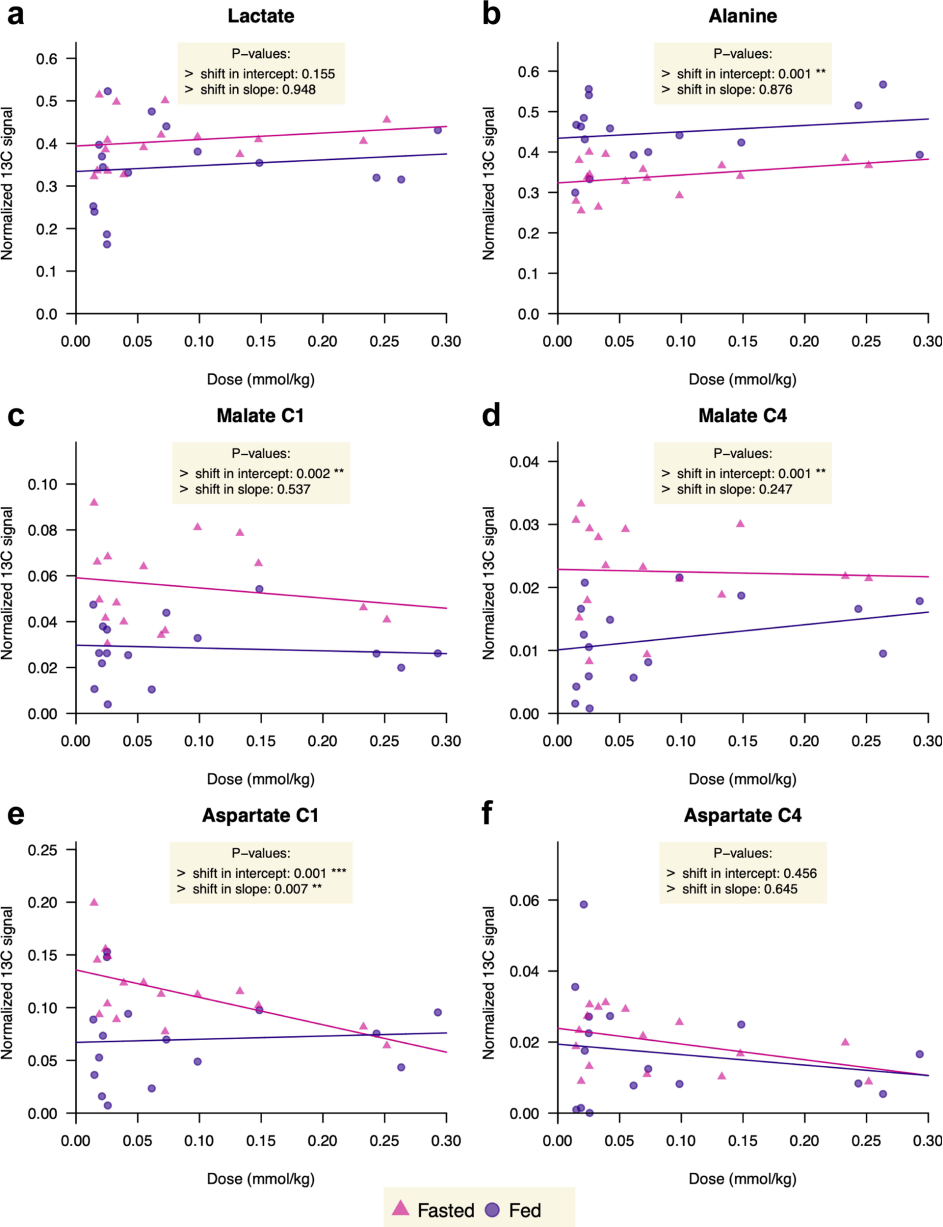
Scatter-plot of the ^13^C-metabolites signal intensities normalized to the total sum of metabolites as a function of the injected dose of HP [1-^13^C]pyruvate. **a** HP [1-^13^C]lactate, (**b**) HP [1-^13^C]alanine, (**c**) HP [1-^13^C]malate, (**d**) HP [4-^13^C]malate, (**e**) HP [1-^13^C]aspartate, and (**f**) HP [4-^13^C]aspartate. Points for 32 experiments (16 fed; 16 fasted) are coded by nutritional state (purple circles: fed; pink triangles: fasted). The fitted regressionabb line of a heteroscedastic linear model is shown for each state. The *p*-values correspond to the two-sided Wald t-tests of equality of intercept and slopes.

The [1-^13^C]aspartate signal also showed a small dose dependence (Fig. 3), but unlike bicarbonate, the relative signal decreased with dose in fasted rats. A modest trend for increased lactate and alanine metabolite fraction with pyruvate dose was apparent, and the conversion to alanine was lower in the fasted rats, with the alanine fraction y-intercept decreasing from 0.43 to 0.32. The normalized [1-^13^C]- and [4-^13^C]malate signals did not show a clear dependence on the dose, but, like the [1-^13^C]aspartate, were higher in the fasted condition.

The C4/C1 ratios for aspartate and malate did not show a dependence on the pyruvate dose or the nutrition state, but with a mean of 0.43 **±** 0.15 for fasted state and 0.44 **±** 0.20 for fed state, the [4-^13^C]malate / [1-^13^C]malate ratios were significantly higher than those of aspartate (0.18 **±** 0.07 for fasted state and 0.18 **±** 0.11 for fed state).

### Effect of PEPCK inhibition

**Fig. 4:**
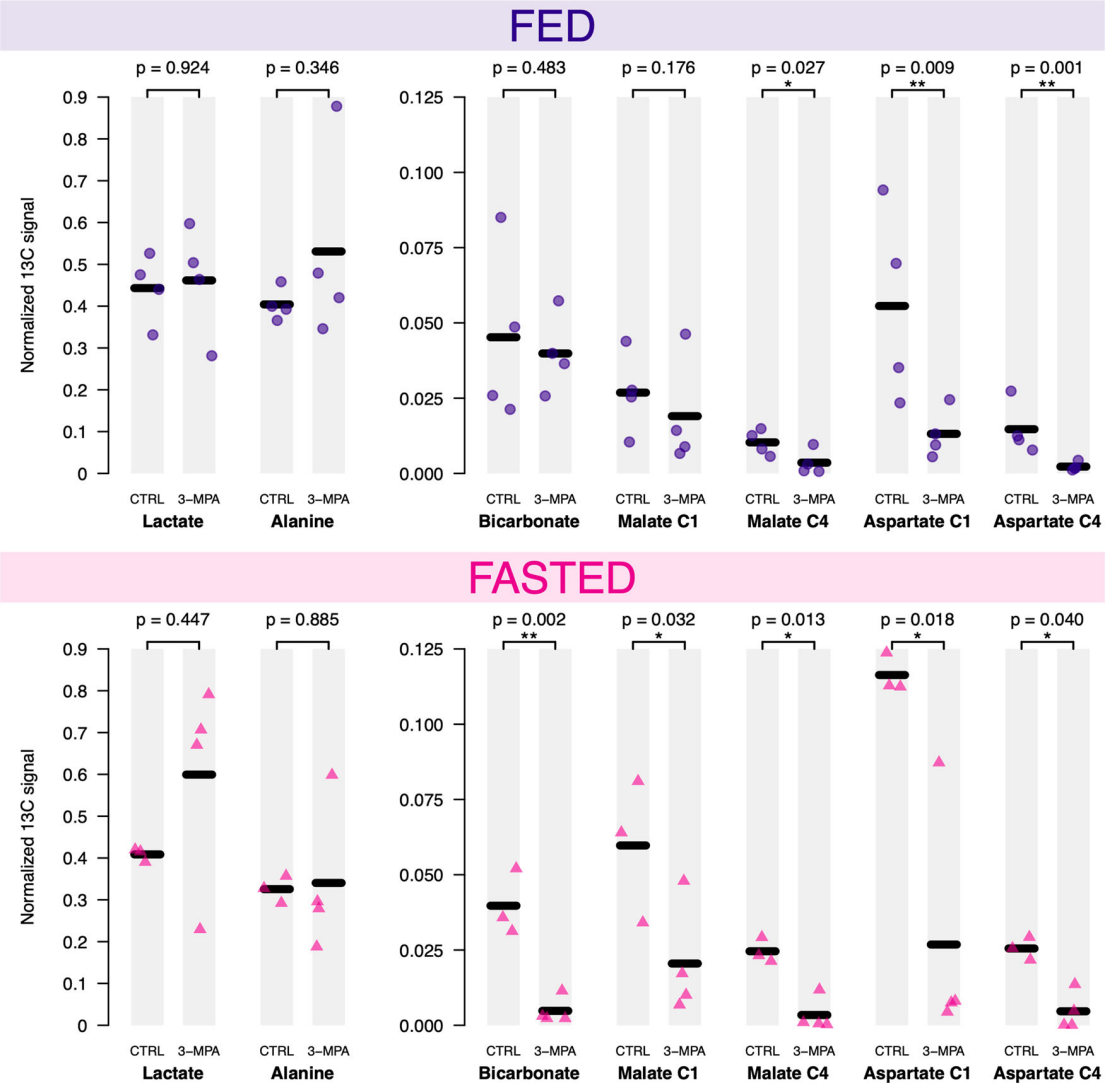
^13^C-metabolites signal intensities in control and PEPCK-inhibited rats in two different nutritional state. Comparison of the HP [1-^13^C]lactate, HP [1-^13^C]alanine, HP [^13^C]bicarbonate, HP [1-^13^C]malate, HP [4-^13^C]malate, HP [1-^13^C]aspartate, and HP [4-^13^C]aspartate signal intensities normalized to the total sum of metabolites measured following the injection of HP [1-^13^C]pyruvate in control and PEPCK-inhibited (3-MPA treated) rats in the fed (upper plot) and fasted (lower plot) state. *P*-values were determined by Student’s *t* tests on the log scale. One-sided tests were performed when analyzing bicarbonate, malate and aspartate outcomes. Two-sided tests were used when analyzing lactate and alanine data. Normalized HP [^13^C]bicarbonate signal intensities show a significant difference between the control and 3-MPA treated groups only in the fasted state.

With decreased PDH flux and increased PEPCK flux expected in the fasted state, the contribution of PEPCK activity to bicarbonate production was tested by treatment with the PEPCK inhibitor 3-MPA administered (100 mg/kg) to fed and fasted rats 1 h prior to the HP [1-^13^C]pyruvate injection. [^13^C]Bicarbonate was almost absent in 3-MPA-treated fasted rats (Fig. 4), consistent with its production by PEPCK. By contrast, 3-MPA had little effect on the [^13^C]bicarbonate signal in the fed state.

Other variations in pyruvate metabolites were also apparent. Aspartate and malate were both lower following 3-MPA treatment (Fig. 4), suggesting decreased PC flux; however, the malate C1 signal in the fed state was not significantly affected, in the fed state. 3-MPA treatment also did not significantly affect the normalized lactate and alanine signals, although bench test experiments confirmed that this treatment resulted in a substantial reduction of blood glucose levels over a 2.5-h period (Fig. S3).

### Effect of fasting and PEPCK inhibition on metabolite levels

To relate the changes in HP [1-^13^C]pyruvate metabolism to the underlying metabolite levels, extracts from liver samples of the fed and fasted untreated and 3-MPA treated rats were analyzed by LC-MS (see Supplementary Methods). Substantial differences in the metabolite profile were apparent between the four conditions (Fig. S4). In particular, the relative levels of aspartate, fumarate and malate were significantly increased with 3-MPA treatment both in fed and fasted rats (Fig. S5), with their signals ranging from 2.3- to 3-fold higher.

## Discussion

In this study, we show that the [^13^C]bicarbonate produced from HP [1-^13^C]pyruvate in the liver of fasted rats is a marker of PEPCK flux. This in vivo finding demonstrates the potential for translating HP MR methods to monitor gluconeogenic metabolism in patients. Additionally, we show that HP ^13^C- pyruvate can be used at low concentrations near its physiological levels to probe multiple aspects of hepatic metabolism, and it reveals differences between fed and fasted rats that may not be apparent when using higher supraphysiological doses.

An earlier study by Janich et al. in non-fasted rats demonstrated varying effects of hyperpolarized [1-^13^C]pyruvate doses of 0.1, 0.2 and 0.4 mmol/kg on its conversion to lactate, alanine and bicarbonate in the liver but did not test near-physiological levels^28^. The current study extends this work to further examine the low-dose regime and the effect of the nutrition state in the liver, particularly with regard to the bicarbonate, aspartate and malate signals. The two studies yield similar results concerning the effect of pyruvate dose on the alanine and fed bicarbonate signals, with no significant change in the former and a strong effect of saturation or feedback inhibition on bicarbonate. The current study tests 4-fold lower doses, and saturation effects are already apparent at 0.1 mmol/kg. The nearly flat profiles of the lactate and alanine curves in Fig. S1 demonstrate that the intracellular ^13^C-label exchange between the [1-^13^C]pyruvate, the [1-^13^C]lactate, and the [1-^13^C]alanine pools is essentially proportional to the dose in both states, and not in a saturating regime with limited uptake. The significant decrease in the normalized [1-^13^C]alanine signal in the fasted state originates from a reduction of the alanine hepatic pool size upon fasting^29^, which agrees with previous HP ^13^C MR studies^21,25^ that used a pyruvate dose of ~1 mmol/kg, or more than 3 times the highest used here confirming the dose independence of the conversion to alanine. However, the dose dependence and greater differences between fed and fasted rats in the conversion of pyruvate to bicarbonate, aspartate and malate seen at the lowest doses tested demonstrate the value in minimizing any metabolic perturbation that may result from a higher dose of a hyperpolarized metabolic probe. Note that although the sufficiently high signal-to-noise ratio (SNR) obtained for the lowest dose injected demonstrates that all metabolites should also be detectable following the injection of a two- to three-fold lower HP [1-^13^C]pyruvate dose to match the basal concentration, the effect of the endogenous pyruvate pool would likely play a greater role in this regime and we could expect greater variability in the apparent metabolism due to the smaller pyruvate fractional ^13^C enrichment.

A key objective of this study was to resolve which enzymes and pathways are responsible for [^13^C]bicarbonate production from HP [1-^13^C]pyruvate in the liver, and the different dose dependence for fed and fasted rats is consistent with PDH flux being the main route of labeled bicarbonate production in the fed state. The regulation of PDH is complex, and pyruvate oxidation in mitochondria shows a U-shaped dependence on pyruvate^30^. Diminishing PDH activity with increasing pyruvate concentrations has been noted in rat liver mitochondria with pyruvate levels greater than ~0.1 mM^31^, similar to the pyruvate dose-dependent response of the normalized [^13^C]bicarbonate signal in the liver of fed rats. The K_m_ obtained from the Michaelis-Menten kinetics analysis is comparable to reported in vitro values^32^, and it is an order of magnitude lower that the typical monocarboxylate transporter-1 (MCT1) K_m_ (~1 mM)^33^, indicating that the observed conversion rate of pyruvate to bicarbonate is not limited by transport into the cell. The lack of a dose dependence of normalized bicarbonate production in the fasted state, on the other hand, is coherent with its being due to PEPCK flux. This result demonstrates the value in using low, near-physiological pyruvate doses, since this difference in bicarbonate signal is most apparent at doses below 0.1 mmol/kg.

The decreased normalized [1-^13^C]aspartate signal with larger pyruvate doses in the fasted state is reminiscent of the lower conversion of HP [1-^13^C]lactate to [1-^13^C]aspartate in the liver recently reported by Chen et al.^34^. There, the dose of injected HP [1-^13^C]lactate was doubled from 0.375 mmol/kg to 0.75 mmol/kg and the normalized aspartate signal decreased by >50% in fasted rats. While this was ascribed to limited flux through PC, that does not appear to be the case here as the conversion to malate is unaffected by dose, as is bicarbonate in the fasted state. In addition to PC flux, the aspartate signal will likely be affected by the turnover rate and redox state as well as transaminase activity and the glutamate /2-oxoglutarate ratio, and they all may be differentially affected by the pyruvate bolus in the fed and fasted states.

3-MPA is an inhibitor of PEPCK that has been shown to induce marked hypoglycemia in both fasted rats^35^ and humans^36^. In the present study, it was used to demonstrate that the [^13^C]bicarbonate signal in fasted animals is largely due to flux through PC and PEPCK. The small, insignificant [^13^C]bicarbonate change in the fed state, along with the significant decrease in aspartate, confirms that PDH flux is the main contributor to the [^13^C]bicarbonate signal in the fed rat liver. Despite the increased hepatic aspartate and malate levels measured by LC-MS, the normalized HP ^13^C aspartate and malate signals were lower following 3-MPA treatment, indicating that the lower signals are likely due to decreased PC flux rather than a decrease in the metabolite pool size. Elevated hepatic levels of malate and aspartate resulting from impaired PEPCK activity have also been reported, with malate increased 10-fold in mice with PEPCK-deficient livers^37^ and malate and aspartate levels respectively 4.9- and 1.4-fold higher in 3-MPA-treated rats^38^. At least two mechanisms may account for the decreased labeling of aspartate and malate. Malate is an inhibitor of PC^39^, and the elevated levels may contribute to the decreased PC flux by feedback inhibition. Additionally, the oxaloacetate pool size will correspondingly increase with malate and aspartate, and this will result in a lower fractional enrichment with the same PC flux, and lower labeling of malate and aspartate.

While the near-absence of conversion of [1-^13^C]pyruvate to bicarbonate in the fasted rat liver following treatment with 3-MPA provides strong evidence of the specificity of the bicarbonate signal for PEPCK flux under these conditions, a number of other factors must be considered in order to relate it to the underlying rate of glucose synthesis. These include the rate of pyruvate cycling mediated by malic enzyme or pyruvate kinase, and the contribution of glycerol to gluconeogenic flux. Moreno et al. have argued that HP [^13^C]bicarbonate does not correlate with the rate of GNG because of the undeterminable amount of pyruvate recycled back to the system via pyruvate kinase^40^. This is certainly possible in well-fed animals, but it has been shown that liver pyruvate cycling flux is only 6% of mitochondrial pyruvate metabolism in overnight-fasted rats^41^. On the other hand, glycerol has been estimated to contribute to one-third of glucose production in fasted mice^42^.

HP [^13^C]bicarbonate in the fasted rat liver is produced from [4-^13^C]oxaloacetate, and its signal is therefore a function of the fractional enrichment at this site, the PEPCK flux and ^13^C polarization level. Both the cytoplasmic and mitochondrial forms of PEPCK may contribute to the observed bicarbonate signal. While cytoplasmic PEPCK is the main form in rodent liver and is required for gluconeogenesis from pyruvate in mice^37^, the mitochondrial form constitutes 2–25% of the PEPCK activity in the rat liver^43–45^, so its contribution to PEPCK-derived bicarbonate, while likely <25%, is not well defined.

**Fig. 5:**
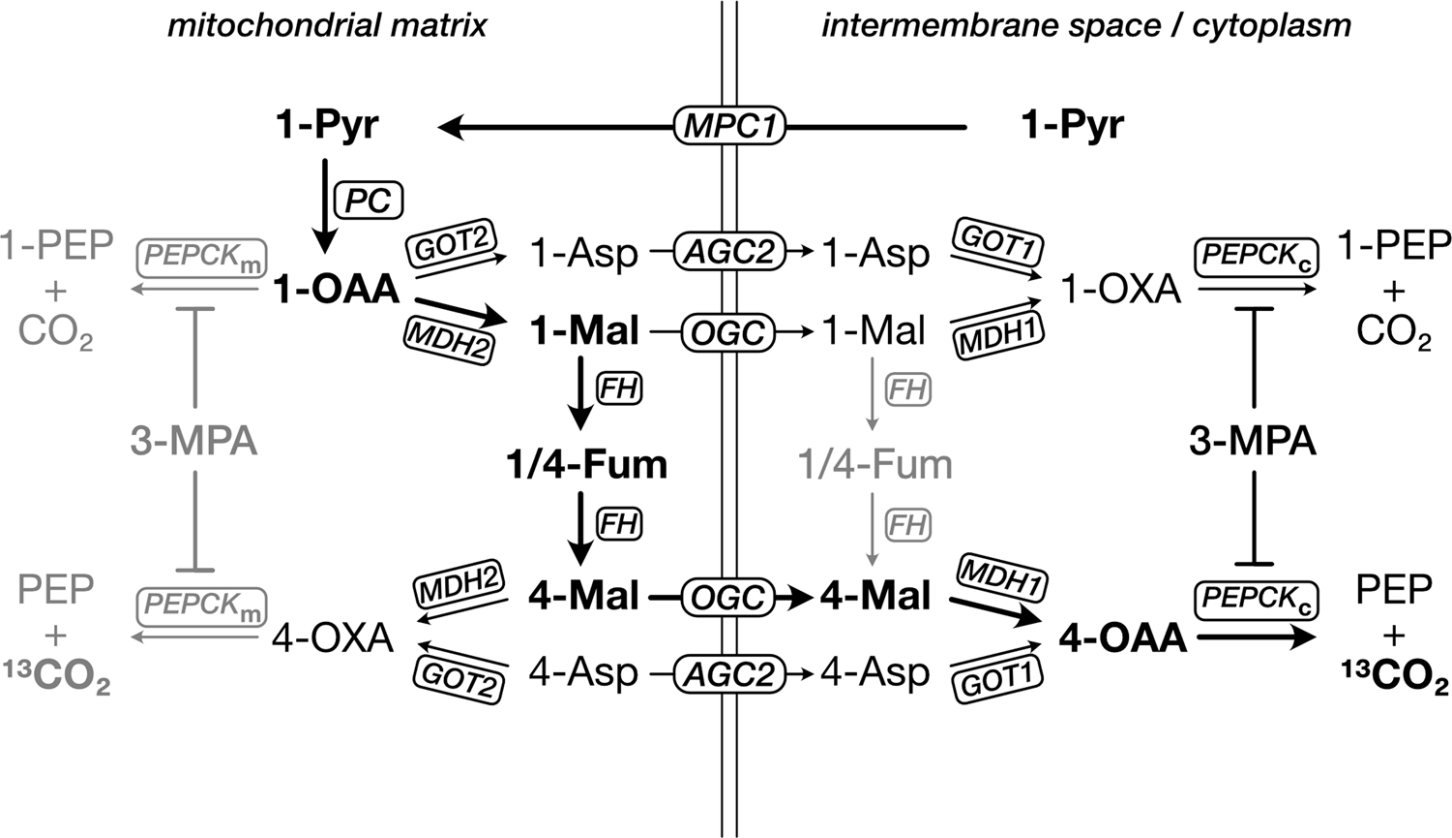
Simplified schematic depicting the route of [1-^13^C]pyruvate (1-Pyr) to [4-^13^C]oxaloacetate (4-OAA) to yield [^13^C]bicarbonate from PEPCK flux. Pyruvate carboxylation takes place in the mitochondrion and transporters of the malate-aspartate shuttle facilitate export to the cytoplasm. Label exchange of malate from the 1- to 4-position is mediated by fumarase (FH). Reversible reactions and transport steps between PC and PEPCK are indicated as unidirectional for simplicity and to illustrate label flow. The main route to cytoplasmic 4-OAA is indicated in bold. MPC1, mitochondrial pyruvate carrier-1; GOT, glutamate oxaloacetate aminotransferase; MDH, malate dehydrogenase; AGC2, aspartate-glutamate antiporter; OGC, 2-oxoglutarate/malate carrier; PEPCK_m_, mitochondrial PEPCK; PEPCK_c_, cytoplasmic PEPCK.

The route of HP [1-^13^C]pyruvate through cytoplasmic PEPCK involves several steps subsequent to carboxylation by PC, engaging the malate-aspartate shuttle to exit the mitochondrion^46^. These routes are summarized in Fig. 5. Conversion to [4-^13^C]oxaloacetate involves four steps in the mitochondrion and either five or seven or steps with export to cytoplasm. By contrast, only three enzymatic steps are required to yield cytoplasmic [1-^13^C]oxaloacetate, which is converted to [1-^13^C]PEP. Oxaloacetate labeled at C1 is therefore the main labeled isotopomer, and the C1 signals of malate and aspartate are more prominent than C4. Given the higher level of fumarase activity in mitochondria^47^, it is likely that most of the exchange of C1 to C4 occurs there. The different C4/C1 ratios for malate and aspartate indicate that their pools are not exchanging rapidly with each other. Any change in these rates of C1-to-C4 label exchange will therefore affect the apparent rate of PEPCK flux assessed by the fasted bicarbonate signal. While we observe no significant change in the ratios with fasting, Merritt et al. did see a decrease in the malate C4/C1 ratio in the perfused fasted mouse liver^17^. While [1-^13^C]PEP is expected to be the main labeled product of PEPCK, it was not observed. With a chemical shift of ~171.9 ppm^48^, the spectral peak of [1-^13^C]PEP is likely obscured by the dominant [1-^13^C]pyruvate signal at 171.1 ppm.

Although a direct relationship between HP pyruvate-to-bicarbonate production in the fasted rat liver and the rate of gluconeogenic flux remains to be demonstrated, some published results suggest such a link. For instance, Lee et al.^20^ report that glucagon treatment increases aspartate and bicarbonate production from HP pyruvate in fasted mouse liver, which is consistent with its induction of increased PC and PEPCK flux and gluconeogenesis.

We demonstrated that the conversion of physiological levels of HP [1-^13^C]pyruvate to malate and aspartate can be detected in the rat liver in vivo, and that PEPCK flux is responsible for the labeled bicarbonate produced in the fasted state. These results therefore show the potential to use HP ^13^C-pyruvate to assess hepatic GNG in fasted patients.

The distance in chemical shift of the [^13^C]bicarbonate signal from the other signals (Fig. 1a, b) minimizes potential interference and is advantageous for translating this technique to humans. Indeed, although this study was performed at high field, which aided the resolution of the aspartate and malate peaks, the [^13^C]bicarbonate peak would be just as easily measured at standard clinical fields of 1.5 T or 3 T. This, together with its dose independence up to 0.29 mmol/kg—3 times the typical dose (0.1 mmol/kg) currently used in HP ^13^C MR clinical studies^16^—demonstrates the potential of hepatic HP ^13^C-pyruvate-to-bicarbonate conversion as a viable clinical biomarker for GNG in fasted subjects. Significantly, we have also shown here the feasibility of detecting all relevant metabolites in vivo with a lower HP ^13^C-pyruvate dose of 0.023 mmol/kg. Higher SNR could also be obtained by using an acquisition scheme based on spectral-spatial pulses optimizing the flip angle for each metabolite would also most likely improve the SNR for specific metabolites such as bicarbonate^49^. The sensitivity of HP ^13^C MR should therefore provide the opportunity to obtain [^13^C]bicarbonate images in the human liver in a way similar to what has been done in the human heart and brain^50,51^. Its applicability to measure hepatic PEPCK flux in human subjects will, however, depend on the degree of [4-^13^C]oxaloacetate production. Additionally, it remains to be determined whether and to what extent the mitochondrial form of PEPCK would also contribute to the PEPCK flux measurements in humans.

## Methods

### Preparation of HP [1-^13^C]pyruvate

Neat ^13^C-labelled pyruvic acid doped with 20 mM OX063 trityl radical (Albeda, Denmark) was polarized in a 7 T custom-built DNP polarizer operating at 1.00 ± 0.05 K using a microwave source set at 196.8 GHz with a nominal output power of 55 mW^52,53^. After 1.5 h, the frozen sample was rapidly dissolved with 6 ml preheated deuterated phosphate buffer (pH ~7.5). The HP ^13^C-pyruvate solution was automatically transferred into a separator/injection pump located inside a 9.4 T, 31 cm horizontal-bore MR magnet (Magnex Scientific, Yarnton, UK) following a previously described protocol^54,55^.

### Animals

All animal procedures were approved by the regional veterinary authorities in Switzerland and experiments were conducted according to their guidelines. Male Sprague Dawley rats (∼200 g) were in two groups, fed and overnight fasted. They were singly housed in cages in a temperature-controlled room with a 12:12-h light-dark cycle. Fed animals had free access to water and standard pellet rat chow (Kliba Nafag 3436; Provimi Kliba, Kaiseraugst, Switzerland). Fasted animals had no access to chow overnight to deplete hepatic glycogen. Animals were anaesthetized with isoflurane (1-2%) in oxygen from the cannulation until the end of the MR experiments. A femoral vein was catheterized for substrate administration, and a femoral artery was catheterized for blood sampling and invasive blood pressure measurements. Blood samples were taken to measure glucose levels 10 min before the injection of HP ^13^C-pyruvate. The body temperature of anesthetized rats inside the magnet was maintained between 37.5 and 38.5 °C with warm water circulating through the animal bed. In all experiments, blood pressure, body temperature and respiratory rate were continuously monitored. No significant physiological changes were observed during the injection. After the experiments, liver tissue samples were collected immediately following sacrifice and rapidly frozen in liquid nitrogen for further analysis by LC-MS.

### PEPCK inhibition

A 0.5 mL aqueous solution of 3-MPA (Toronto Research Chemicals, Canada) at a dose of 100 mg/kg of body weight was prepared. 0.4 M NaOH was added to the preparation to balance the acidity to physiological pH. The solution was administered via intraperitoneal (IP) injection one hour prior to the HP [1-^13^C]pyruvate infusion for minimal metabolic perturbation.

### Verification of inhibitor infusion protocol

The physiological effects of 3-MPA were tested in a bench experiment with an overnight-fasted animal (450 g). The inhibitor injection protocol was identical as mentioned above. Respiration and temperature of the animal were monitored continuously. 100 μl-blood samples were collected from the tail vein before the injection and then every 30 min over 2.5 h. Blood glucose and plasma lactate levels were measured using automated glucose/lactate analyzers (Reflotron Plus, Roche, Basel, Switzerland and GM7 Micro-Stat, Analox Instruments, London, UK).

### MR acquisitions

The rat was placed inside the magnet holder in supine position with a custom-built quadrature ^1^H/single loop ^13^C surface coil placed on top of its shaved liver region. The exact position of the coil relative to the liver was adjusted and verified with ^1^H images acquired using a gradient echo sequence (8 slices, slice thickness = 2 mm, TR = 50 ms, TE = 3 ms, field of view = 30 × 30 mm, Resolution= 128 × 128, flip angle = 60°). The static magnetic field was homogenized for a selected voxel to reduce the proton linewidth using the FASTESTMAP protocol. A 1 ml bolus of HP [1-^13^C]pyruvate solution at various concentrations was injected through the femoral vein over ~9 s. In vivo ^13^C MR spectra were recorded with a repetition time of ~3 s using a VNMRS spectrometer (Varian, Palo Alto, CA coupled to the 9.4 T horizontal-bore magnet. 30° BIR4 adiabatic RF excitation pulses were applied, and spectra were acquired with proton decoupling. All imaging, shimming and ^13^C MRS acquisitions were respiration gated and cardiac triggered. For the PEPCK inhibition study with 3-MPA administration, fed and fasted rats were injected with 0.066 ± 0.007 mmol/kg of HP [1-^13^C]pyruvate and compared to untreated rats receiving a comparable dose.

### Data analysis

The exact concentration of the injected [1-^13^C]pyruvate solution was determined at the end of each ^13^C MRS experiment by NMR analysis on a 400 MHz spectrometer (Bruker BioSpin SA, Fällanden, Switzerland). The ^13^C-pyruvate signal intensity of a ∼200 μL sample of residual solution collected from inside the separator/injection pump was compared to a ^13^C-urea internal standard of known concentration. The concentration of the injected ^13^C-pyruvate dose and the rat blood volume estimated from the body weight^56^ were used to calculate the initial blood concentration. Note that a body density of 1 kg/L was assumed to convert mmol/kg to mM for the Michaelis-Menten kinetics analyses. The metabolite peak integrals were quantified and normalized with a total ^13^C signal obtained from summed spectra analyzed using VNMRJ (Agilent Technologies, Santa Clara, CA). The number of individual spectra included in each summed spectrum was determined using the fixed condition that the SNR of the last spectrum from each series of acquisitions has to be higher than 5. The summed spectra were obtained using equal weight for each individual spectrum in the time series. Although each metabolite might have a different T_1_, this will not affect the relative comparisons between different metabolic states or dose effects performed in this study.

### Statistics and reproducibility

Student’s *t* tests were used to compare the mean of the fractions of sum of metabolite signals of the control and PEPCK-inhibited groups for each outcome interest for fed and fasted rats. One-sided tests were performed when analyzing bicarbonate, malate and aspartate outcomes. Two-sided tests were used when analyzing lactate and alanine data. Sensitivity analyses using Welch’s tests on the original data yielded similar conclusions. The evolution of each outcome as a function of the injected pyruvate dose was modeled by means of linear models with dose, treatment group and their interaction as predictors. Both model checks and likelihood ratio tests comparing homoscedastic and heteroscedastic models suggested the presence of greater residual variance for low-dose levels given the predictors, so that heteroscedastic linear models were used^57^. The log scale was preferred for bicarbonate. Wald t-tests on the fitted models were used to test for the equality of intercepts and slopes between conditions. Estimates of the two-parameter Michaelis–Menten model per group were obtained by means of the function drc() of the R package drm. All authors had access to the study data and have reviewed and approved the final manuscript.

### Reporting summary

Further information on research design is available in the Nature Research Reporting Summary linked to this article.

## Supplementary information

Supplementary Information

Description of Additional Supplementary Files

Supplementary Data 1

Reporting Summary

## Data Availability

Source data for the figures are given in Supplementary Data 1 and any remaining info can be requested from the corresponding author upon reasonable request.
