# Supplementary material for: [13C]bicarbonate labelled from hyperpolarized [1-13C]pyruvate is an in vivo marker of hepatic gluconeogenesis in fasted state
Source: Commun Biol. 2022 Jan 10;5:10. doi: 10.1038/s42003-021-02978-2 (PMC8748681; doi:10.1038/s42003-021-02978-2)
Supplement: Supplementary file 1 — Supplementary Information [file 42003_2021_2978_MOESM1_ESM.pdf]

Supplementary Information

[^{13}C]bicarbonate labelled from hyperpolarized [1-^{13}C]pyruvate is an in vivo marker of hepatic gluconeogenesis in fasted state

Emine Can[1], Jessica A.M. Bastiaansen[1,2], Dominique-Laurent Couturier[3], Rolf Gruetter[1], Hikari A.I. Yoshihara[1*], Arnaud Comment[3,4*]

[1]Institute of Physics, Ecole Polytechnique Fédérale de Lausanne, CH-1015 Lausanne, Switzerland

[2]Department of Diagnostic and Interventional Radiology, Lausanne University Hospital and University of Lausanne, Lausanne, Switzerland

[3]Cancer Research UK Cambridge Institute, University of Cambridge, Cambridge, Cambridgeshire CB2 0RE, United Kingdom

[4]General Electric Healthcare, Chalfont St Giles, Buckinghamshire HP8 4SP, United Kingdom

*These authors contributed equally

This supplementary information includes Figures S1-S5 as well as the supplementary methods.

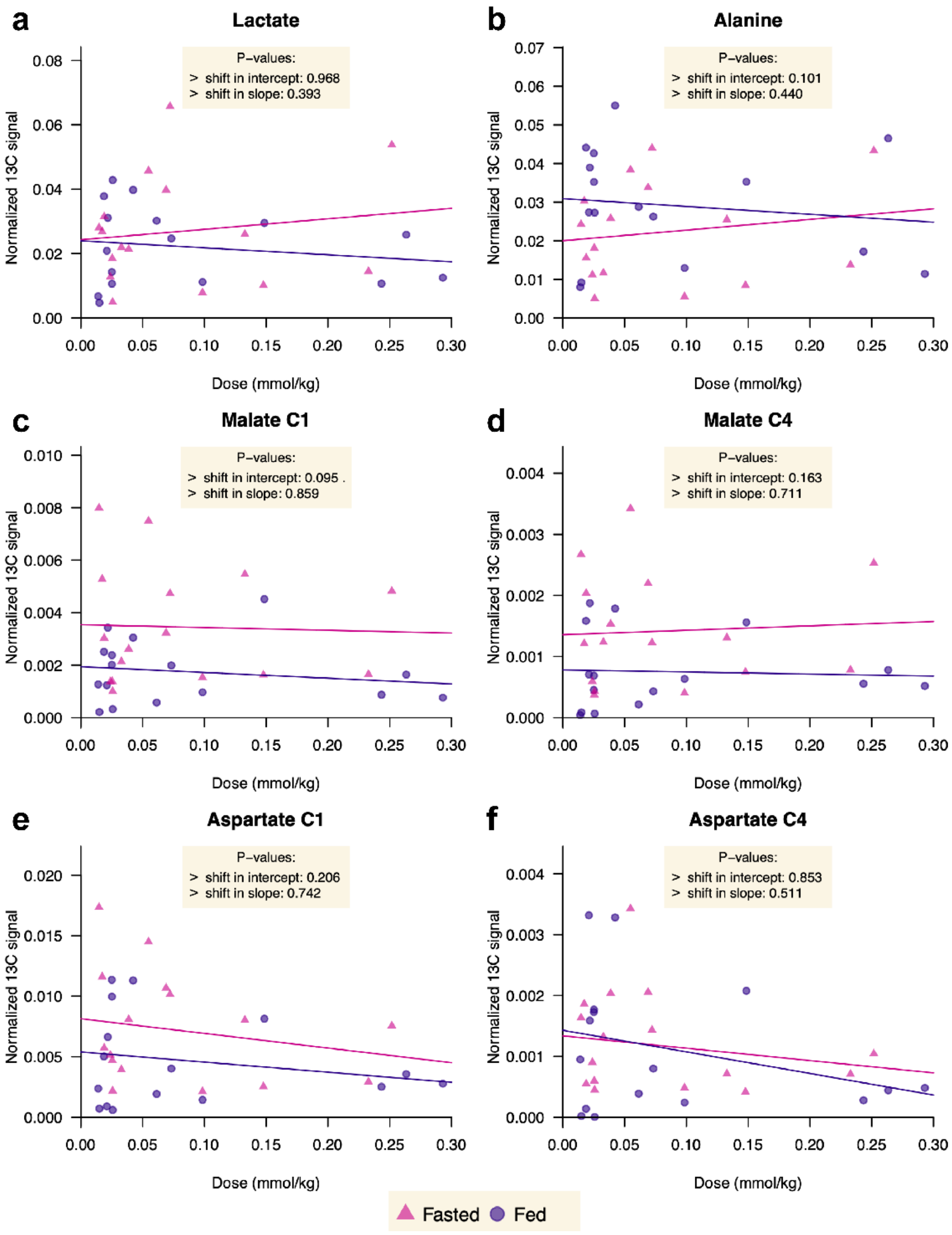

Figure S1. Scatter-plot of the ^{13}C-metabolites signal intensities normalized to the total ^{13}C signal as a function of the injected dose of HP [1-^{13}C]pyruvate. (a) HP [1-^{13}C]lactate, (b) HP [1-^{13}C]alanine, (c) HP [1-^{13}C]malate, (d) HP [4-^{13}C]malate, (e) HP [1-^{13}C]aspartate, and (f) HP [4-^{13}C]aspartate. Points for 32 experiments (16 fed; 16 fasted) are coded by nutritional state (purple circles: fed; pink triangles: fasted). The fitted regression line of a heteroscedastic linear model is shown for each state. The p-values correspond to the two-sided Wald t-tests of equality of intercept and slopes.

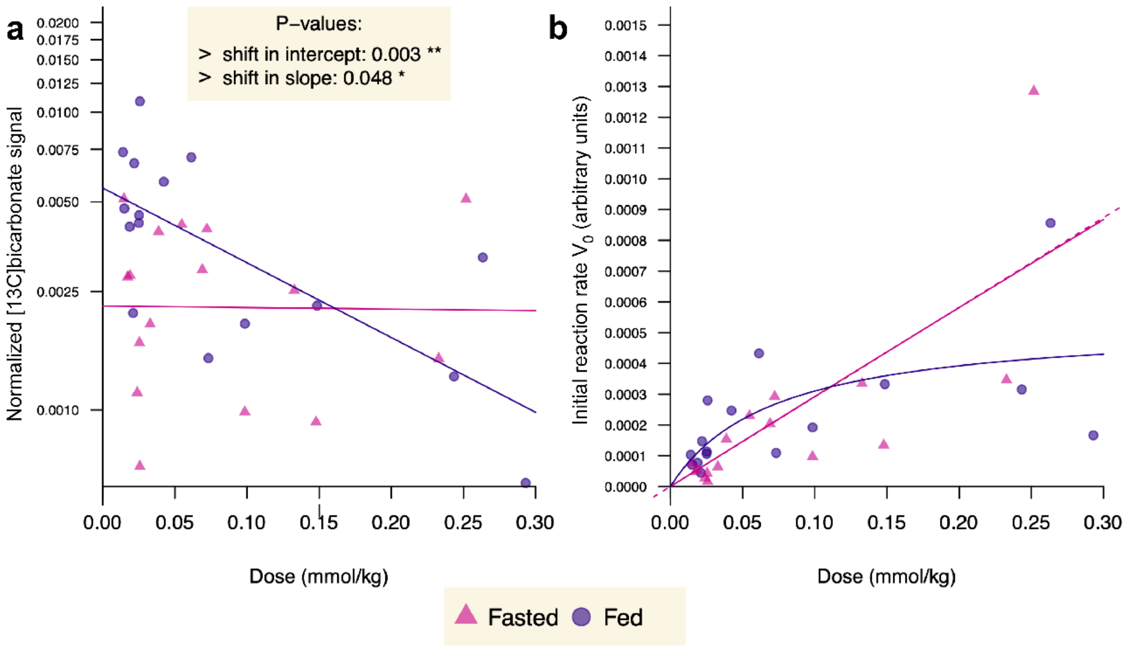

Figure S2. Scatter-plot of the HP [^{13}C]bicarbonate signal intensities normalized to the total ^{13}C signal and corresponding initial reaction rate as a function of the injected dose of HP [1-^{13}C]pyruvate. Points for 32 experiments (16 fed; 16 fasted) are coded by nutritional state (purple circles: fed; pink triangles: fasted). **a.** The fitted regression line of a heteroscedastic linear model is shown for each state. The p-values correspond to the two-sided Wald t-tests of equality of intercept and slopes. **b**. The initial reaction rate V_0 (in arbitrary units) was obtained by multiplying the normalized HP [^{13}C]bicarbonate signal, which can be assumed to be proportional to an apparent pyruvate-to-bicarbonate conversion rate constant ($k_{pyr\text{-}bic}$), with the corresponding substrate dose. The fit with a Michaelis-Menten model yields a K_m value of 0.072 ± 0.063 mM for the fed state and K_m = 17 ± 67 mM for the fasted state, the latter being clearly better fit with a linear regression (dotted line).

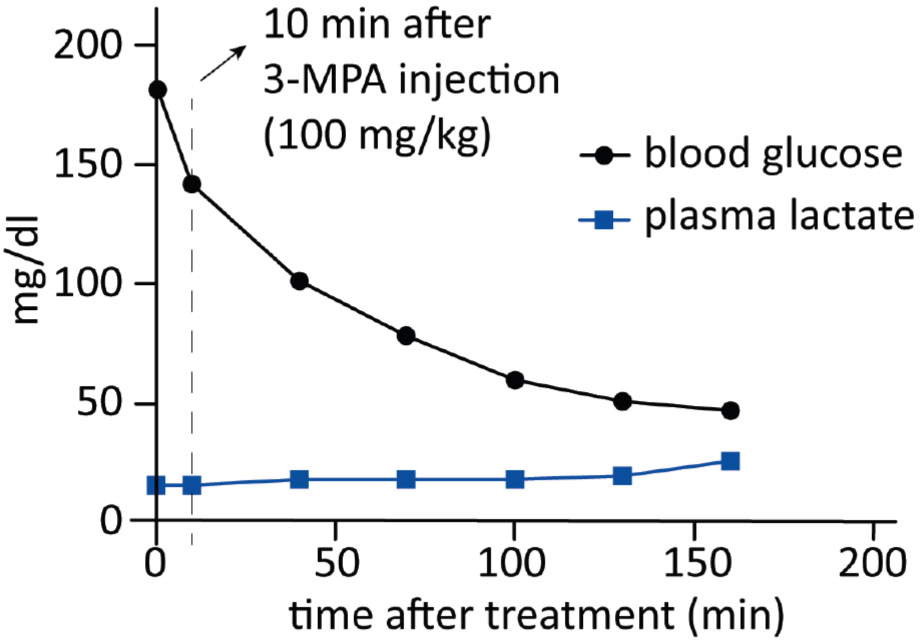

Figure S3. **Hypoglycemic effect of 3-mercaptopicolinic acid treatment in an overnight fasted rat**.

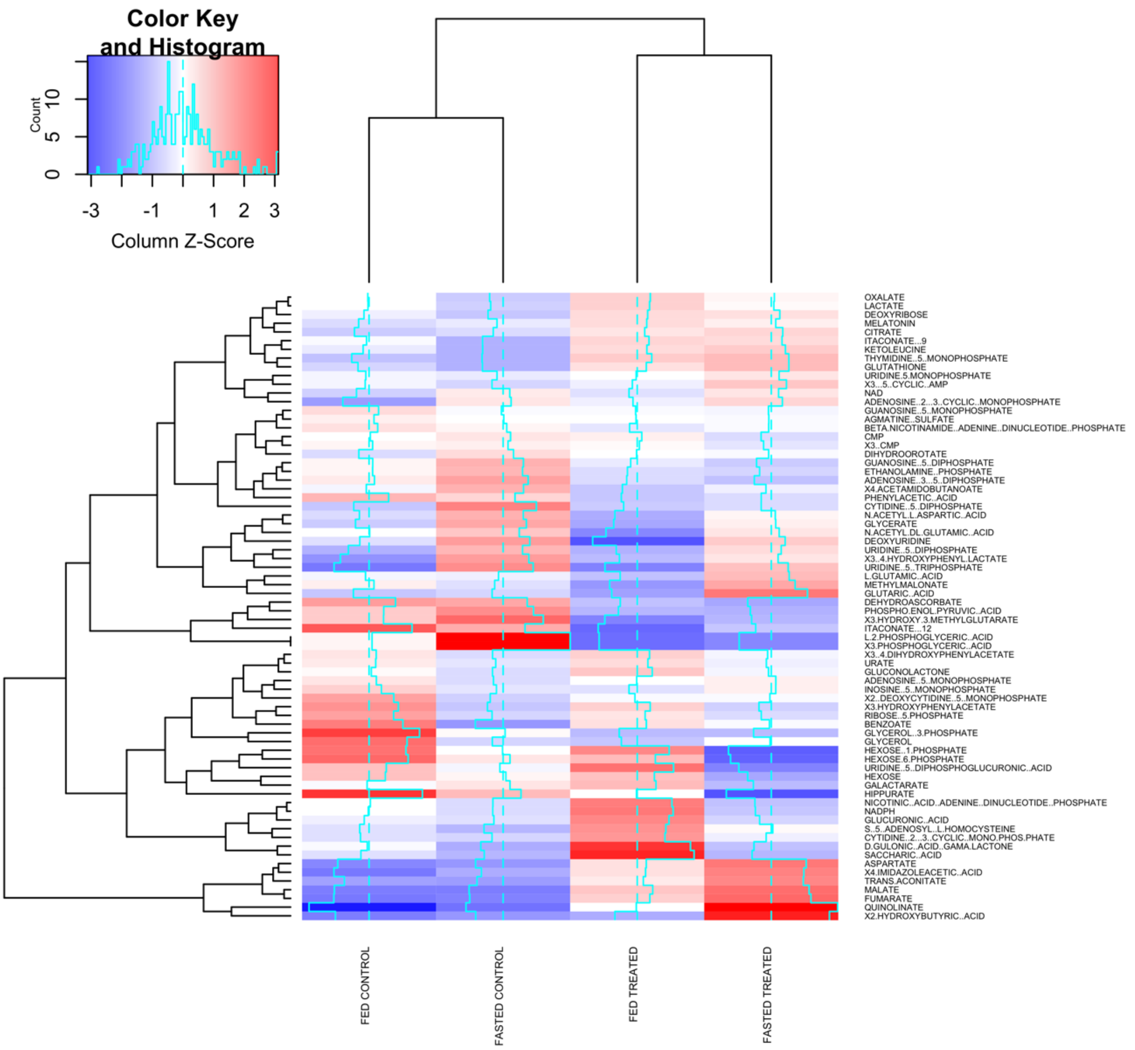

Figure S4. **Heatmap of liver metabolite profile differences.** Organic acids in extracts of liver samples from fasted (3) and fed (4) control and fasted (4) and fed (3) 3-MPA treated rats were quantitated by LC-MS. Mean normalized metabolite signal for each group are expressed as the ratio to the mean metabolite signal across all groups.

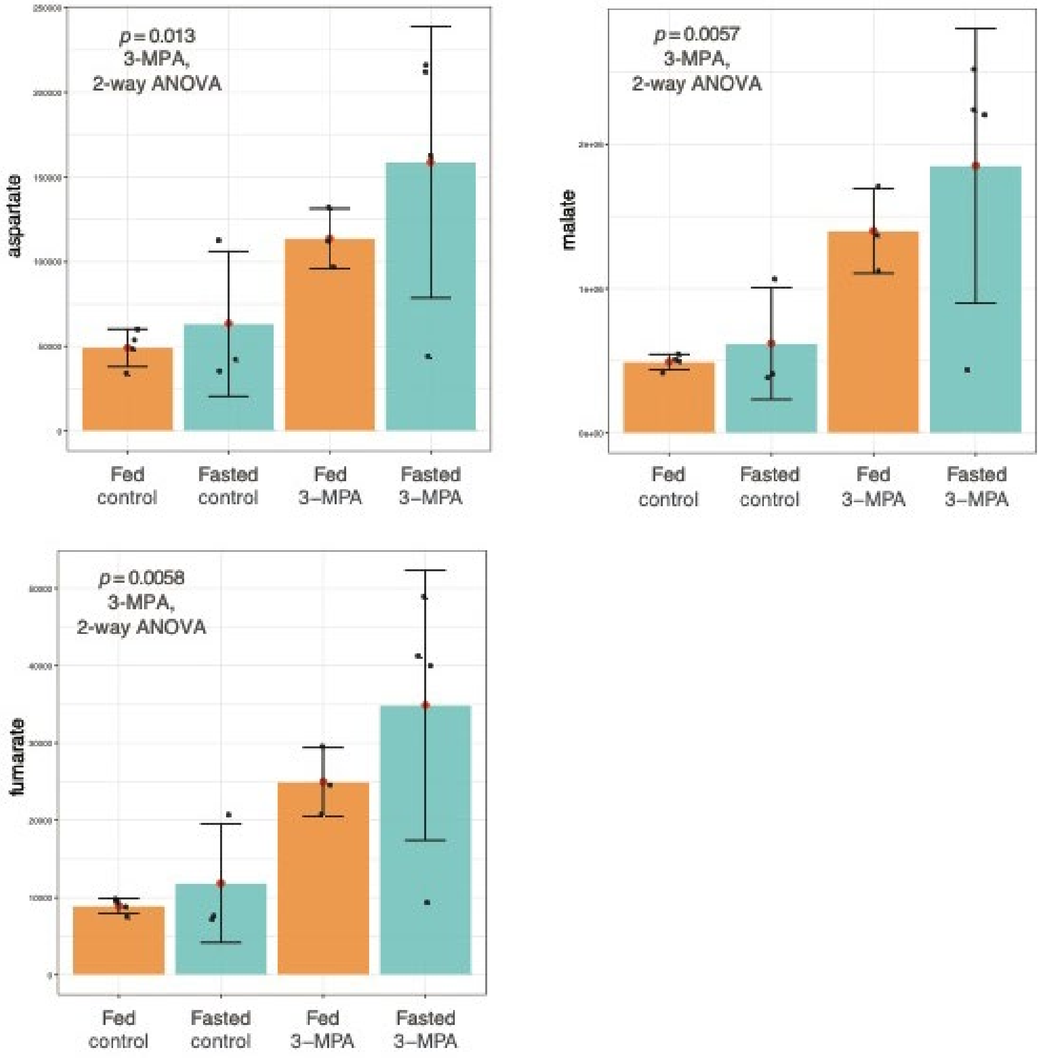

Figure S5. **Elevated liver aspartate, malate and fumarate levels are detected in 3-MPA treated rats.** Signal intensities are in arbitrary units, normalized by tissue extract protein concentration. Error bars represent the standard deviation. *P* values from 2-way ANOVA are with respect to the effect of 3-MPA treatment.

Supplementary methods

Analysis of metabolomic data. Metabolite signals from LC-MS analysis of organic acids in liver tissue extracts were normalized by the protein concentration to provide relative quantitation. For each metabolite, the mean signal for each condition were normalized to the mean signal across all conditions, and these ratios were used to generate a heatmap (heatmap.2) in R (v. 4.0.2), with clustering of metabolites following the same trends. Two-way ANOVA analyses of the aspartate, malate and fumarate signals were performed in R.
